# Quercetin Ameliorates Comorbid Insomnia in Diarrhea-Predominant Irritable Bowel Syndrome via the PI3K/AKT/NF-κB Signaling Pathway

**DOI:** 10.3390/biomedicines14030692

**Published:** 2026-03-17

**Authors:** Guangming Liu, Xiangpan Kong, Yiru Zhao, Nianshan Cai, Haiyi Wang, Hongxu Sun, Peng Zhao

**Affiliations:** 1 Wuxi School of Medicine, Jiangnan University, 1800 Lihu Avenue, Wuxi 214122, China; 6222809120@stu.jiangnan.edu.cn (G.L.); x.kong@jiangnan.edu.cn (X.K.); 6232811004@stu.jiangnan.edu.cn (Y.Z.); 6232811001@stu.jiangnan.edu.cn (N.C.); 6242811002@stu.jiangnan.edu.cn (H.W.); 2 MOE Medical Basic Research Innovation Center for Gut Microbiota and Chronic Diseases, Wuxi School of Medicine, Jiangnan University, 1800 Lihu Avenue, Wuxi 214122, China; 3 Department of Traditional Chinese Medicine (Internal Medicine), School Hospital, Jiangnan University, Wuxi 214062, China; sunhongxu336@jiangnan.edu.cn

**Keywords:** chronic insomnia disorder, irritable bowel syndrome with diarrhea, gut–brain axis, quercetin, PI3K/AKT signaling pathway, network pharmacology

## Abstract

**Background:** Chronic insomnia disorder (CID) frequently coexists with diarrhea-predominant irritable bowel syndrome (IBS-D), a comorbidity characterized by gut–brain axis dysfunction and persistent inflammatory activation. However, the molecular mechanisms underlying this overlap remain incompletely understood, and effective multitarget interventions are lacking. **Objectives:** This study aimed to identify quercetin as a potential bioactive compound for IBS-D-associated insomnia and to investigate whether its protective effects are associated with modulation of the PI3K/AKT/NF-κB signaling pathway. **Methods:** CID- and IBS-D-related targets were collected from public databases. Candidate compounds were screened using bioinformatics and network pharmacology analyses, followed by molecular docking. Experimental validation was conducted in 36 male C57BL/6J mice assigned to control, CID+IBS-D model, quercetin-treated, and quercetin-plus-Recilisib-treated groups. Sleep-related behavior, EEG/EMG-derived sleep architecture, intestinal function, inflammatory markers, and pathway-related proteins were assessed. **Results:** Quercetin was identified as a core candidate compound. Network pharmacology revealed 43 shared targets among CID, IBS-D, and quercetin, with significant enrichment in PI3K/AKT-related signaling. In vivo, quercetin improved sleep-associated phenotypes and intestinal dysfunction; reduced visceral hypersensitivity; restored ZO-1 and Occludin expression; suppressed hypothalamic and colonic inflammatory responses; and was accompanied by reduced phosphorylation of PI3K, AKT, IκB, and NF-κB p65 in the hypothalamus. Quercetin also increased hypothalamic 5-HT1A and GABA_A Rα5 expression. These effects were partially reversed by Recilisib, supporting the involvement of PI3K/AKT-associated signaling in quercetin-mediated protection. **Conclusions:** Quercetin alleviated key sleep-related and IBS-D-like phenotypes in a composite murine model of gut–sleep comorbidity. The protective effects were associated with reduced inflammatory activation and modulation of PI3K/AKT/NF-κB-related signaling. These findings support quercetin as a promising candidate for gut–brain axis-related comorbid disorders, while further studies are needed to define pathway specificity, tissue exposure, and translational applicability.

## 1. Introduction

Insomnia is a widespread public health issue, reflecting the increasing burden of stress-related and psychosomatic disorders in modern society, with a global prevalence of approximately 16.2% [1]. Chronic Insomnia Disorder (CID) not only impairs cognitive and emotional functions but also exhibits bidirectional interactions with various systemic diseases [2]. Among these comorbidities, irritable bowel syndrome (IBS) has attracted particular attention due to its high co-occurrence with insomnia. Epidemiological studies indicate that 37.6% of individuals with insomnia also experience IBS symptoms [3], while IBS affects about 11.2% of the global population [4]. The diarrhea-predominant subtype of IBS (IBS-D) is notably associated with sleep disturbances. Dysregulation of the gut–brain axis plays a pivotal role in IBS-D by contributing to visceral hypersensitivity, emotional imbalance, and sleep fragmentation, which exacerbate both gastrointestinal and sleep-related symptoms [3,5]. This reciprocal relationship not only worsens disease severity but also leads to increased healthcare utilization and reduced quality of life. Current pharmacotherapies for insomnia and IBS focus mainly on symptom management, targeting neurotransmission, gastrointestinal motility, or sleep–wake regulation, but they provide only transient relief, with fewer than half of patients achieving sustained improvement [6,7]. Additionally, the shared neuroimmune mechanisms underlying both conditions remain insufficiently addressed, underscoring the need for integrated, multitarget approaches that can modulate both peripheral and central dysfunctions.

Recent research highlights the gut–brain–microbiota axis as a critical communication network linking the neural, endocrine, and immune systems. Dysregulation of this axis, marked by systemic inflammation and neuroimmune activation, appears to be a common pathological mechanism in both insomnia and IBS-D [8,9]. However, the specific molecular pathways linking these disorders remain unclear. Bioinformatic tools, including differential gene expression and weighted gene co-expression network analysis (WGCNA), offer effective methods for identifying key molecular targets and regulatory networks in complex diseases [10]. These approaches hold promise for uncovering novel therapeutic strategies for comorbid conditions.

Bioactive compounds derived from natural sources have garnered growing attention due to their multitarget pharmacological potential. Quercetin (QU), a flavonoid found in many fruits and vegetables, exhibits potent anti-inflammatory, antioxidant, and neuroprotective properties [11,12]. Quercetin has been shown to modulate gut barrier integrity, immune balance, and neural excitability, suggesting its ability to restore homeostasis via regulation of the gut–brain axis. These attributes make quercetin a promising candidate for the treatment of comorbid insomnia and IBS-D, both of which involve neuroimmune and inflammatory dysregulation.

Despite promising preclinical findings, the molecular basis of quercetin’s bidirectional modulation of intestinal and neural pathways remains poorly defined. Understanding its multitarget effects may provide valuable insights into the integrated management of gut–brain disorders. Therefore, the present study aims to explore the therapeutic potential of quercetin in a comorbid IBS-D and insomnia model by identifying shared molecular mechanisms and signaling pathways. Through bioinformatic analysis combined with experimental validation, this research seeks to clarify how natural bioactive compounds exert multitarget effects on complex comorbidities, advancing the understanding of gut–brain axis mechanisms and facilitating the development of holistic therapeutic strategies in line with modern pharmacology.

## 2. Materials and Methods

### 2.1. Dataset Selection, Preprocessing, and DEG Detection

This study analyzed differentially expressed genes (DEGs) in chronic insomnia (CI) and diarrhea-predominant irritable bowel syndrome (IBS-D) using microarray datasets GSE208668 and GSE36701, retrieved from the NCBI GEO database (https://www.ncbi.nlm.nih.gov/geo/, accessed on 6 March 2024). The datasets were normalized and processed using the GEOquery package in R (v2.66.0) [13]. Differential expression analysis was performed using the limma package (version 4.4.1) [14], and DEGs were identified based on *p*-values and log-fold changes (GSE208668: *p*-value < 0.05, LogFC > 1; GSE36701: *p*-value < 0.05, LogFC > 0.1). Quantile normalization was applied to reduce technical variance, and duplicates were removed, with missing values imputed using the dplyr package (v1.1.4) [15]. Venn diagrams were generated to illustrate overlapping gene sets, and heatmaps were produced using the pheatmap (v1.0.12) and ggplot2 packages (v3.5.1) [16].

### 2.2. Gene Co-Expression Network Construction

Weighted gene co-expression network analysis (WGCNA) (v1.72.5) was employed to investigate the relationship between phenotypes and genes by analyzing the GSE208668 and GSE36701 datasets in R software (v4.2.3) [10]. The data were converted into adjacency matrices for clustering to identify module eigengenes (MEs). The overlap between CID and IBS was visualized using Venn diagram packages (v1.7.3), and modules were merged and clustered based on MEs.

### 2.3. Drug–Gene Interaction

Candidate therapeutic compounds targeting shared DEGs between chronic insomnia and IBS-D were predicted using Enrichr and the DSigDB database (https://maayanlab.cloud/Enrichr/, accessed on 18 December 2024 ) [17]. The gene–drug interaction network was visualized using Cytoscape (3.10.3) [18].

### 2.4. Disease and Drug Interaction Target Prediction

Pharmacological targets of quercetin (QU) were identified using the TCMSP database (https://www.tcmsp-e.com/load_intro.php, accessed on 24 March 2024.). Disease-related gene targets for chronic insomnia (CI) and IBS-D were retrieved from the DisGeNET (https://disgenet.com/, accessed on 25 March 2024), GeneCards (https://www.genecards.org/, accessed on 25 March 2024), and OMIM (https://www.omim.org/, accessed on 25 March 2024) databases. The intersection of compound and disease targets was analyzed using Venn diagram analysis to identify potential therapeutic targets [19].

### 2.5. Enrichment Analysis Using GO and KEGG

Gene Ontology (GO) and Kyoto Encyclopedia of Genes and Genomes (KEGG) enrichment analyses were conducted using the DAVID database (https://davidbioinformatics.nih.gov/, accessed on 1 April 2024), with subsequent visualization in R (v4.2.3) and Cytoscape (v3.10.3) [20].

### 2.6. Protein–Protein Interaction (PPI) Network Analysis

Protein interactions in Homo sapiens were analyzed using the STRING database [21], with a minimum confidence score of 0.40. The PPI network was constructed in Cytoscape (v3.10.3), and five hub genes were identified using the cytoNCA and cytoHubba algorithms, providing insights into potential therapeutic targets and associated disease pathways [18].

### 2.7. Molecular Docking

Molecular docking was performed to predict ligand–receptor interactions using the CDOCKER protocol in Discovery Studio 2022 (v22.1). Target protein structures (e.g., PI3Kα and AKT1–3) were obtained from the Protein Data Bank (PDB), and quercetin (QU) was retrieved from PubChem. Docking simulations and result visualizations were performed using Discovery Studio, following established procedures as described by Liu et al. (2023) [22].

### 2.8. Animal Procedures and Pharmaceutical Preparation

Sixty-three male C57BL/6 mice (6–8 weeks old, 20–25 g) were purchased from SPF (Suzhou, China) Biotechnology Co., Ltd. (License No. SCXK(Su)2022-0006). The animals were housed under specific-pathogen-free (SPF) conditions at the Animal Center of Jiangnan University. Before the experiment, the mice were acclimatized for one week under controlled conditions (12 h light/dark cycle, 22–25 °C, 40–60% humidity) with free access to standard chow and water. Drug administration by oral gavage was performed using curved 8F gavage needles. Senna granules (Jiangsu Aidi Pharmaceutical Co., Ltd. Yangzhou, China) and quercetin (Shanghai Meryer Biochemical Technology Co., Ltd., Shanghai, China) were prepared as aqueous suspensions according to the manufacturers’ instructions. Recilisib (MedChemExpress, Monmouth Junction, NJ, USA) and sodium pentobarbital (Sigma-Aldrich, St. Louis, MO, USA) were administered intraperitoneally. All animal procedures were approved by the Experimental Animal Welfare and Ethics Committee of Jiangnan University (Approval No. JN 20240115c0600308[024]). All animals that completed the experimental protocol were included in the final analysis, and no animals were excluded after group allocation. Predefined humane exclusion criteria included severe illness, inability to feed or drink, or body weight loss exceeding 20%; however, none of the animals met these criteria during the study.

### 2.9. Animal Grouping and Modeling

After one week of acclimatization, male C57BL/6 mice were randomly assigned to experimental groups (*n* = 9 per group). A comorbid insomnia–diarrhea-predominant irritable bowel syndrome (IBS-D) model was established using a composite protocol integrating sleep deprivation, restraint stress, and senna granule gavage. Chronic sleep deprivation (6 h/day) was applied to induce insomnia-like phenotypes and neuroimmune dysregulation associated with central nervous system disturbance [23,24,25], while repeated restraint stress (2 h/day for 3 weeks) was used to activate stress-response pathways and disrupt sleep architecture [25,26]. To induce IBS-D-like intestinal dysfunction, mice subsequently received senna leaf gavage (0.607 g/mL) for 2 weeks, an approach commonly used to provoke diarrhea-like symptoms, visceral hypersensitivity, and intestinal dysfunction relevant to IBS-D [27,28,29,30]. The combined stress–senna paradigm has been reported to reproduce behavioral and intestinal alterations relevant to diarrhea-predominant IBS and stress-related comorbidity [27,28]. In the present study, model validity was evaluated based on multidimensional phenotypic changes, including diarrhea-like stool output, visceral hypersensitivity, metabolic disturbances, sleep fragmentation, and fatigue-like manifestations, as previously described in experimental IBS-D and insomnia models [25,29,30]. Quercetin (purity ≥ 97%, CAS: 117-39-5; Shanghai Meryer Chemical Technology Co., Ltd., Shanghai, China) was administered once daily by oral gavage at a dose of 100 mg/kg during the intervention period.

In the second experimental phase, an additional intervention group was established in which Recilisib (10 mg/kg, intraperitoneally) was administered once daily for 1 week to CID + QU mice, thereby generating a CID + QU + Recilisib group (*n* = 9). All other groups and treatments remained identical to those used in the phenotypic validation phase. In the phenotypic validation phase, three groups were included: control (CON), model (CID), and quercetin-treated (CID + QU) mice. For mechanistic validation, the fourth group (CID + QU + Recilisib) was added to probe pathway involvement. This design allowed separation of overall phenotypic effects from pathway-specific mechanistic interrogation. All procedures were performed according to standardized protocols to ensure reproducibility and minimize systematic bias [31,32]. Detailed procedures for model construction and experimental group assignment are shown in Figure S1 (Supplementary Figure S1). Only male mice were used in this exploratory mechanistic study to reduce biological variability during the initial pathway-focused evaluation; however, this may limit the generalizability of the findings to both sexes.

### 2.10. Evaluation of Metabolic Activity and General Behavioral Observation

General health status and behavioral phenotypes of mice were assessed throughout the experimental period by daily observation. Parameters include mental state, spontaneous activity, grooming behavior, fur condition, posture, and social interaction. Animals were monitored daily for general health status, body weight, grooming behavior, posture, and activity levels. Humane endpoints were predefined as severe distress, persistent immobility, or body weight loss exceeding 20%, at which point animals would be humanely euthanized. No animals reached humane endpoint criteria during the study.

### 2.11. Positive Reflex Test for Sodium Pentobarbital Loss of Righting Reflex (LORR)

Sleep latency and sleep duration were evaluated using a sodium pentobarbital-induced loss-of-righting reflex (LORR) test. Sodium pentobarbital (45 mg/kg) was administered intraperitoneally, and the time to loss and recovery of the righting reflex was recorded [25,33].

### 2.12. Stool Characteristics (Bristol Score) and Abdominal Withdrawal Reflex (AWR)

Fecal characteristics were assessed using the Bristol Stool Form Scale, a validated scoring system for evaluating stool consistency and morphology [29,34]. After 18 h of fasting, an A6-F catheter coated with liquid paraffin was inserted 1.5 cm into the anus under isoflurane anesthesia. The balloon was inflated sequentially to volumes of 0.25, 0.35, 0.50, and 0.65 mL at 5 min intervals. Visceral pain sensitivity was evaluated using the AWR scoring system as previously described [30,35].

### 2.13. Electroencephalography (EEG)/Electromyography (EMG) Monitoring

EEG and EMG electrodes were implanted under tribromoethanol anesthesia as previously described [32]. Postoperatively, the surgical site was disinfected with iodophor, and animals were rewarmed using a warming blanket. Animals were closely monitored during recovery and received appropriate perioperative care to minimize discomfort. After a recovery and adaptation period, EEG/EMG signals were recorded using the Spike2 system and analyzed with Lunion Stage software (v2.2) [33].

### 2.14. Western Blotting (WB)

Protein extraction, electrophoresis, immunoblotting, and densitometric analysis were performed as previously described, with minor modifications. Briefly, proteins from colonic and hypothalamic tissues were extracted using RIPA lysis buffer. After SDS–PAGE separation and transfer onto PVDF membranes, the blots were incubated with the following primary antibodies: GABAA receptor α5 (Rabbit mAb, SAB, Greenbelt, MD, USA, Cat#49791, 1:1000), 5-HT1A receptor (Rabbit pAb, SAB, Cat#41456, 1:1000), Occludin (Rabbit pAb, Proteintech, Rosemont, IL, USA, Cat#27260-1-AP, 1:5000), ZO-1 (Rabbit pAb, Proteintech, Cat#21773-1-AP, 1:5000), Phospho-PI3K (Tyr467/Tyr199) (Rabbit pAb, Zen-Bio, Chengdu, China, Cat#341468, 1:1000), PI3K p85α (Rabbit mAb, Zen-Bio, Cat#R22768, 1:1000), Phospho-AKT (Ser473) (Rabbit pAb, Zen-Bio, Cat#381555, 1:1000), AKT1 (Rabbit pAb, Zen-Bio, Cat#342529, 1:1000), Phospho-IκBα (Ser32/36) (Rabbit pAb, Zen-Bio, Cat#340776, 1:1000), IκBα (Rabbit pAb, Zen-Bio, Cat#380662, 1:1000), Phospho-NF-κB p65 (Ser536) (Rabbit pAb, Zen-Bio, Cat#310013, 1:1000), NF-κB p65 (Rabbit mAb, Zen-Bio, Cat#310013, 1:1000), and β-actin (internal control, Rabbit pAb, SAB, Cat#41456, 1:10,000). Membranes were then incubated with appropriate HRP-conjugated secondary antibodies. Protein bands were visualized using ECL reagents and quantified using ImageJ software (v1.54p).

### 2.15. RNA Isolation, Reverse Transcription, and Quantitative Polymerase Chain Reaction (RT-PCR)

Total RNA was extracted from the colonic and hypothalamic tissues using Trizol reagent (Beyotime Biotechnology, Shanghai, China). cDNA synthesis was performed with the qPCR + gDNA wiper HiScript II Q RT SuperMix (Vazyme Biotech Co., Ltd., Nanjing, China). Amplification was conducted by using the ChamQ Universal SYBR qPCR Master Mix (Vazyme Biotech Co., Ltd.) on the Step-One Plus system (Thermo Fisher Scientific, Waltham, MA, USA). The primers targeted TNF-α (forward: 5′-CTGTGAAGGGAATGGGTGT-3′; reverse: 5′-CAGGGAAGAATCTGGAAAGG-3′), IL-1β (forward: 5′-CCAGCTTCAAATCTCACAGCAG-3′; reverse: 5′-CTTCTTTGGGTATTGCTTGGGATC-3′), IL-6 (forward: 5′-CCACCAAGAACGATAGTCAA-3′; reverse: 5′-TTTCCACGATTTCCCAGA-3′), IL-10 (forward: 5′-AGCCCACCAAGAACGATA-3′; reverse: 5′-GGTTGTCACCAGCATCAG-3′), and GAPDH (forward: 5′-TCTCGAATGACTCCTCCACTC-3′ and reverse 5′-AAGCTCCGCCTGGTAAT-3′). All primers were synthesized by Sangon Biotech (Shanghai) Co., Ltd. (Shanghai, China) [36].

### 2.16. Outcome Measures

Outcome measures assessed in this study included behavioral, electrophysiological, and molecular endpoints. Behavioral outcomes included body weight, food and water intake, stool consistency (Bristol Stool Scale), visceral sensitivity (abdominal withdrawal reflex), and sleep latency and duration assessed by the loss-of-righting reflex test. Electrophysiological outcomes comprised EEG/EMG-derived sleep architecture parameters. Molecular and inflammatory outcomes included hypothalamic neurotransmission-related proteins (5-HT_1_A and GABA_ARα5), intestinal barrier proteins (ZO-1 and Occludin), PI3K/AKT/NF-κB pathway activation, and inflammatory cytokine expression (TNF-α, IL-1β, IL-6, and IL-10) in hypothalamic and colonic tissues.

This study was designed as an exploratory mechanistic investigation; therefore, no single primary outcome measure was predefined for sample size determination. Instead, treatment effects were evaluated based on the internal consistency of multiple independent outcomes across behavioral, electrophysiological, and molecular domains.

### 2.17. Statistical Analysis

Statistical analyses were performed using GraphPad Prism version 10.1.2 (GraphPad Software, San Diego, CA, USA). Data are presented as mean ± SEM. Normality of data distribution was assessed using the Shapiro–Wilk test. For normally distributed data, comparisons between two groups were performed using unpaired two-tailed Student’s *t*-tests, while comparisons among multiple groups were conducted using one-way or two-way analysis of variance (ANOVA), followed by Tukey’s post hoc test where appropriate.

For data that did not meet normality assumptions, nonparametric tests were applied, including the Mann–Whitney U test for two-group comparisons and the Kruskal–Wallis test followed by Dunn’s multiple comparisons test for multiple-group analyses. Repeated-measures data were analyzed using two-way ANOVA with repeated measures when applicable. A *p* value < 0.05 was considered statistically significant.

## 3. Results

### 3.1. General Behavioral and Phenotypic Observations

In addition to quantitative measurements, general behavioral and phenotypic conditions were systematically observed. Mice in the control (CON) group exhibited normal locomotor activity, smooth and glossy fur, frequent social interactions, and normal postural behavior, indicating a stable and healthy general condition. In contrast, mice in the CID model group showed markedly reduced spontaneous activity, disorganized and dull fur, decreased social interaction, and an increased incidence of abnormal postural behaviors, reflecting an overall deterioration in behavioral and phenotypic status. Compared with the CID group, quercetin-treated mice demonstrated partial to significant improvement in overall behavioral condition. These mice displayed increased locomotor activity, enhanced social interaction, and reduced abnormal postural behaviors, suggesting an improvement in general health status.

### 3.2. Identification of DEGs and Module–Trait-Related Interacting Genes in CID and IBS-D

**Figure 1:**
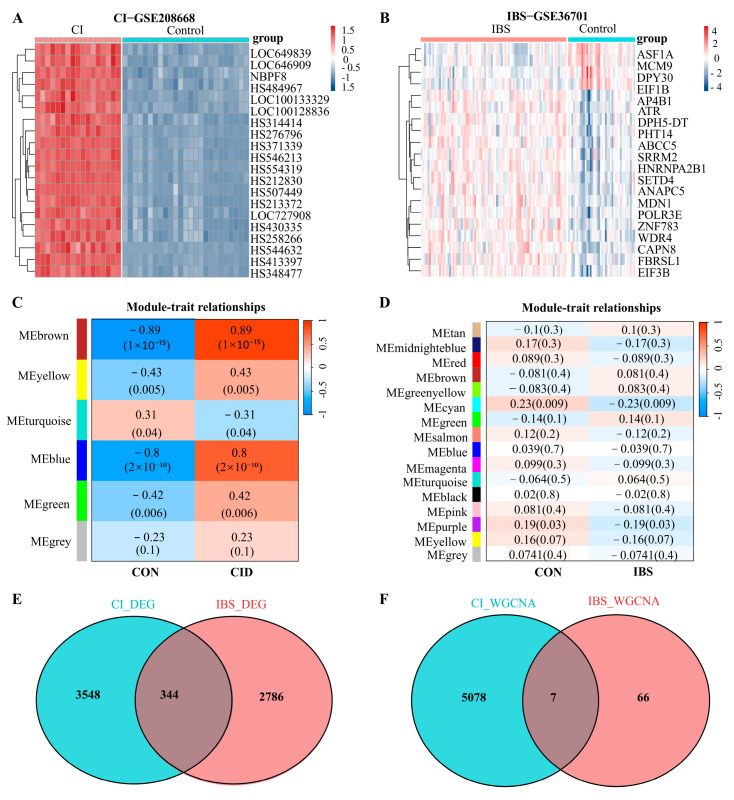
Differential expression analysis and weighted gene co-expression network analysis of CID and IBS-D. (**A**) Heatmap showing the differentially expressed genes (DEGs) in the CID group (GSE208668) compared with the control group. (**B**) Heatmap showing the DEGs in the IBS-D group (GSE36701) compared with the control group. (**C**) Module–trait correlation analysis for CID, illustrating the associations between gene co-expression modules and CID-related phenotypes. (**D**) Module–trait correlation analysis for IBS-D, illustrating the associations between gene co-expression modules and IBS-D–related phenotypes. (**E**) Venn diagram showing the overlapping DEGs shared between CID and IBS-D. (**F**) Venn diagram showing the overlapping gene modules identified by weighted gene co-expression network analysis (WGCNA) in the CID and IBS-D datasets.

Gene expression profiles of individuals with CID and IBS-D, along with their corresponding control groups, were analyzed using the GSE208668 and GSE36701 datasets. A total of 3892 differentially expressed genes (DEGs) were identified in the GSE208668 dataset, while 3130 DEGs were detected in GSE36701 (Supplementary Figure S2). Heatmaps illustrating the top 20 significantly differentially expressed genes in each dataset are shown in Figure 1A,B. Venn diagram analysis was subsequently performed to identify overlapping DEGs between CID and IBS-D, resulting in the identification of 344 shared genes (Figure 1E; Supplementary Table S1).

Weighted gene co-expression network analysis (WGCNA) was then conducted to construct gene co-expression networks associated with CID and IBS-D and to examine correlations between gene modules and disease traits. The soft-thresholding powers were set at 18 for CID and 5 for IBS-D (Supplementary Figure S3). Among the disease-associated modules identified, quercetin-related targets were enriched in the most significant modules. In the CID dataset, the MEblue (r = 0.89, *p* = 1 × 10^−10^) and MEbrown (r = 0.89, *p* = 1 × 10^−15^) modules exhibited the strongest correlations with clinical traits. In the IBS-D dataset, the MEcyan (r = 0.23, *p* = 0.009) and MEpurple (r = 0.19, *p* = 0.03) modules showed the most significant associations (Figure 1C,D). Subsequent Venn diagram analysis identified seven overlapping genes shared between CID- and IBS-D–related modules (Figure 1F; Supplementary Table S2).

### 3.3. Identification of Potential TCM Monomers Based on Insomnia-Related Genes in Comorbid Conditions

To identify potential therapeutic compounds associated with comorbid insomnia, overlapping trait-related module genes obtained from the previous analyses (Figure 1E,F) were combined and deduplicated. This procedure resulted in a total of 351 candidate genes, which may be involved in the shared molecular mechanisms underlying chronic insomnia disorder (CID) (Supplementary Table S3).

Subsequently, enrichment analysis was performed using the DSigDB database to assess statistical significance and gene–drug binding scores. Based on the enrichment results, the top 50 medicinal compounds with statistically significant *p*-values were initially identified. After excluding redundant entries, 43 small-molecule compounds with potential therapeutic relevance to CID were retained (Supplementary Table S4).

**Figure 2:**
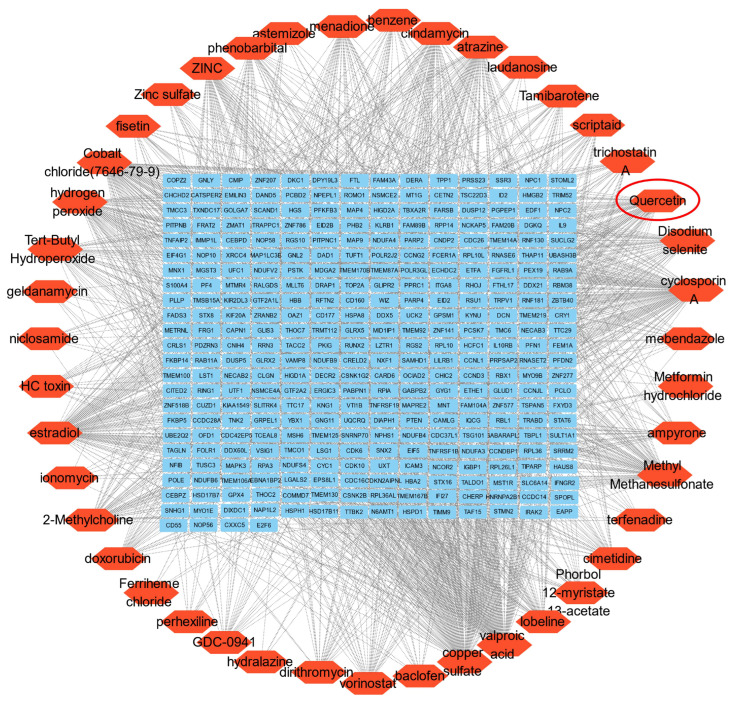
Identification of genes and candidate Chinese herbal monomers associated with comorbid insomnia. Gene–drug interaction network illustrating the relationships between candidate genes and predicted therapeutic compounds. Green–blue rounded rectangles represent genes, whereas orange hexagons denote compounds. The red circle highlights quercetin, the sole Chinese herbal monomer among the predicted compounds. Based on its significance as a representative natural product, it was selected for subsequent experimental validation.

Among these candidate compounds, the traditional Chinese medicine monomer quercetin (QU) was identified as a key candidate due to its strong association with the shared gene set (Figure 2).

### 3.4. Quercetin Improves Sleep Architecture and Intestinal Barrier Dysfunction in Mice with Comorbid Insomnia

To evaluate the therapeutic effects of quercetin (QU) in comorbid insomnia, a mouse model incorporating sleep deprivation, restraint stress, and senna leaf administration was established. Key outcome measures included changes in body weight, food and water intake, visceral sensitivity, stool characteristics, sleep parameters, electroencephalographic activity, and the expression of intestinal barrier– and neurotransmission-related proteins.

#### 3.4.1. Effects of QU on Body Weight and Metabolic Parameters

**Figure 3:**
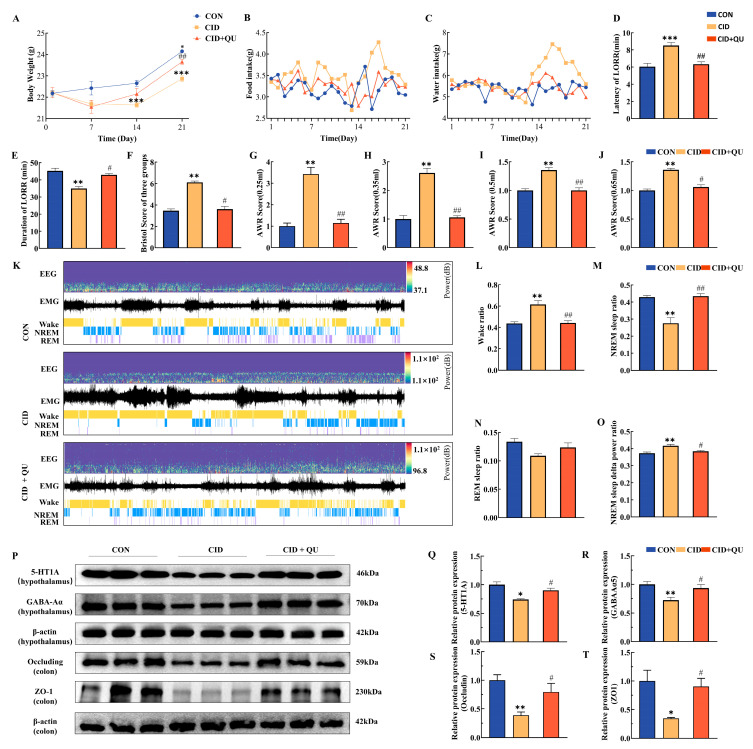
Effects of quercetin on physiological, behavioral, and neurobiological parameters in mice with comorbid insomnia. (**A**) Body weight changes across experimental groups. (**B**) Average food intake over 21 days. (**C**) Average water intake over 21 days. (**D**) Sleep latency is measured by the loss-of-righting reflex (LORR) test. (**E**) Sleep duration was assessed by LORR recovery time. (**F**) Bristol Stool Scale scores. (**G**–**J**) Abdominal withdrawal reflex (AWR) scores under colorectal distension volumes of 0.25, 0.35, 0.50, and 0.65 mL. (**K**) Representative EEG and EMG traces illustrating sleep–wake states, *n* = 3. (**L**) Wake–sleep ratio. (**M**) NREM sleep ratio. (**N**) REM sleep ratio. (**O**) Delta power ratio during NREM sleep. (**P**) Representative Western blot images of 5-HT_1_A, GABA Rα5, Occludin, ZO-1, and β-actin in the CON, CID, and CID + QU groups. (**Q**–**T**) Quantification of relative protein expression levels. Mean ± SEM, *n* = 6. Compared with CON: * *p* < 0.05, ** *p* < 0.01, *** *p* < 0.001; compared with the CID group: ^#^ *p* < 0.05, ^##^ *p* < 0.01. Compared with CID + QU group: ^※^ *p* < 0.05.

Both time and treatment exerted significant effects on body weight, with a significant interaction between these factors. On day 14, mice in the CID group exhibited a significantly lower body weight compared with the CON group (21.63 ± 0.29 g vs. 22.65 ± 0.44 g, *p* < 0.001). By day 21, mice in the QU-treated group showed a significantly higher body weight than those in the CID group (CID + QU: 23.66 ± 0.45 g vs. CID: 22.87 ± 0.29 g, *p* < 0.01) (Figure 3A). Analysis of food and water intake revealed that mice in the CID group consumed more food and water than those in the CON group, whereas QU treatment resulted in intermediate intake levels between the two groups (Figure 3B,C).

#### 3.4.2. Effects of QU on Sleep Parameters

In the loss-of-righting reflex (LORR) test, significant differences in sleep latency were observed among groups. The CID group exhibited a markedly prolonged sleep latency compared with the CON group (8.52 ± 0.33 min vs. 6.05 ± 0.39 min, *p* < 0.001). QU treatment significantly reduced sleep latency relative to the CID group (6.33 ± 0.29 min vs. 8.52 ± 0.33 min, *p* < 0.01), restoring values close to those of the CON group (Figure 3D).

Recovery duration also differed significantly among groups. The CID group showed a shorter recovery time compared with the CON group (34.95 ± 1.15 min vs. 45.25 ± 1.52 min, *p* < 0.01). QU treatment significantly prolonged recovery duration compared with the CID group (42.94 ± 0.79 min vs. 34.95 ± 1.15 min, *p* < 0.05), with no significant difference relative to the CON group (Figure 3E).

#### 3.4.3. Effects of QU on Intestinal Function and Visceral Sensitivity

Bristol Stool Scale scores were significantly elevated in the CID group compared with the CON group, indicating diarrhea-like symptoms. QU treatment significantly reduced these scores toward control levels (Figure 3F).

Abdominal withdrawal reflex (AWR) scores differed significantly among groups across all colorectal distension volumes (0.25–0.65 mL). The CID group exhibited markedly higher AWR scores than the CON group (*p* < 0.01), whereas QU treatment significantly reduced AWR scores compared with the CID group (*p* < 0.01) (Figure 3G–J).

#### 3.4.4. Effects of QU on EEG/EMG-Derived Sleep Architecture

EEG/EMG analysis revealed a significantly higher arousal frequency in the CID group compared with the CON group (0.62 ± 0.04 vs. 0.44 ± 0.02, *p* < 0.01). QU treatment significantly reduced arousal frequency relative to the CID group (0.44 ± 0.02 vs. 0.62 ± 0.04, *p* < 0.01) (Figure 3K,L). The proportion of non-rapid eye movement (NREM) sleep was significantly reduced in the CID group compared with the CON group (0.28 ± 0.03 vs. 0.43 ± 0.01, *p* < 0.01). QU treatment significantly increased NREM sleep duration relative to the CID group (0.44 ± 0.01 vs. 0.28 ± 0.03, *p* < 0.01) (Figure 3M). No significant differences in REM sleep duration were observed among groups (Figure 3N). Analysis of NREM delta power revealed elevated slow-wave activity in the CID group compared with the CON group (0.42 ± 0.01 vs. 0.37 ± 0.01, *p* < 0.01). QU treatment significantly reduced delta power relative to the CID group (0.38 ± 0.00 vs. 0.42 ± 0.01, *p* < 0.05), approaching control levels (Figure 3O).

#### 3.4.5. Effects of QU on Neurotransmitter and Intestinal Barrier Protein Expression

Western blot analysis demonstrated that hypothalamic 5-HT_1_A receptor expression was significantly reduced in the CID group compared with the CON group (0.741 ± 0.011 vs. 1.000 ± 0.051, *p* < 0.05). QU treatment significantly increased 5-HT_1_A expression relative to the CID group (0.902 ± 0.037 vs. 0.741 ± 0.011, *p* < 0.05) (Figure 3P,Q). Similarly, GABA_ARα5 expression was markedly reduced in the CID group compared with the CON group (0.723 ± 0.055 vs. 1.000 ± 0.053, *p* < 0.01), whereas QU treatment significantly increased its expression relative to the CID group (0.937 ± 0.059 vs. 0.723 ± 0.055, *p* < 0.05) (Figure 3R). In colonic tissues, the expression levels of the tight junction proteins Occludin and ZO-1 were significantly reduced in the CID group (*p* < 0.01 and *p* < 0.05, respectively). QU treatment significantly increased the expression of both proteins compared with the CID group (*p* < 0.05 for both), although levels remained below those observed in the CON group (Figure 3S,T). Collectively, these results demonstrate that QU treatment ameliorated multiple pathological features of the comorbid insomnia model. QU improved sleep-related parameters, reduced visceral hypersensitivity, enhanced intestinal barrier integrity, and partially restored the expression of key neural and intestinal proteins associated with comorbid insomnia.

### 3.5. Network Pharmacology and Molecular Docking Identify PI3K/AKT-Associated Targets of Quercetin in Comorbid Insomnia with IBS-D

**Figure 4:**
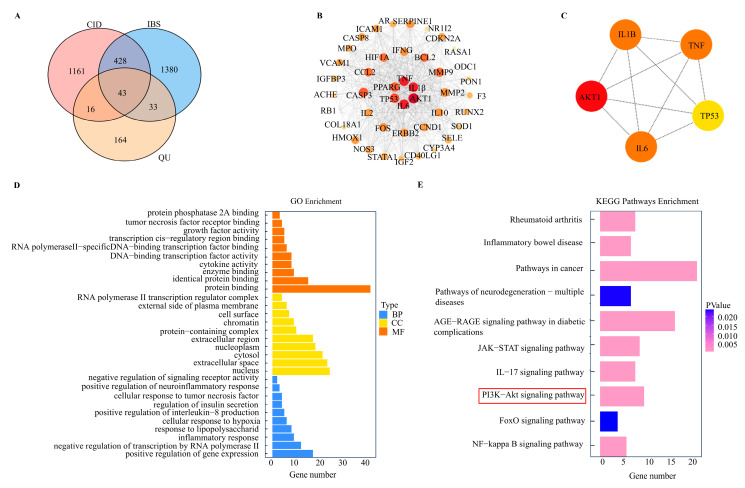
Network pharmacology analysis of quercetin-associated targets in comorbid insomnia with IBS-D. (**A**) Venn diagram showing the overlap of gene targets associated with CID, IBS-D, and quercetin (QU). (**B**) Protein–protein interaction (PPI) network constructed from the overlapping targets.The color gradient from yellow to red represents increasing degree values in the PPI network. (**C**) Hub genes identified using cytoHubba and cytoNCA algorithms. (**D**) Gene Ontology (GO) enrichment analysis of the common driver genes. (**E**) KEGG pathway enrichment analysis of the common driver genes. The red box highlights the PI3K-Akt signaling pathway (*p* = 3.42 × 10^−5^).

To explore the potential molecular targets of quercetin (QU) in comorbid insomnia with IBS-D, disease-related genes were retrieved from the DisGeNET and GeneCards databases, while compound-related targets of QU were obtained from the TCMSP database. Overlapping targets associated with CID, IBS-D, and QU were identified through integrative analysis, yielding a putative network. Venn diagram analysis revealed 43 common driver genes (CDGs) shared among CID, IBS-D, and QU (Figure 4A).

Protein–protein interaction (PPI) network analysis of the 43 CDGs revealed a network consisting of 43 nodes and 555 edges (Figure 4B). Using the cytoHubba and cytoNCA algorithms, several hub genes were identified, including AKT1, IL6, TNF, IL1B, and TP53 (Figure 4C; Supplementary Table S5).

Gene Ontology (GO) enrichment analysis demonstrated that the CDGs were significantly involved in biological processes related to inflammatory response, positive regulation of gene expression, cellular response to hypoxia, TNF-mediated signaling, and neuroinflammatory regulation. These genes were mainly localized to the nucleus, cytoplasm, and protein complexes, and were enriched in molecular functions associated with protein and enzyme binding, cytokine activity, growth-factor activity, and TNF receptor binding (Figure 4D). KEGG pathway enrichment analysis further revealed significant enrichment in the PI3K/AKT signaling pathway, as well as pathways related to inflammatory bowel disease, rheumatoid arthritis, cancer, and neurogenesis (Figure 4E; Supplementary Table S6).

**Figure 5:**
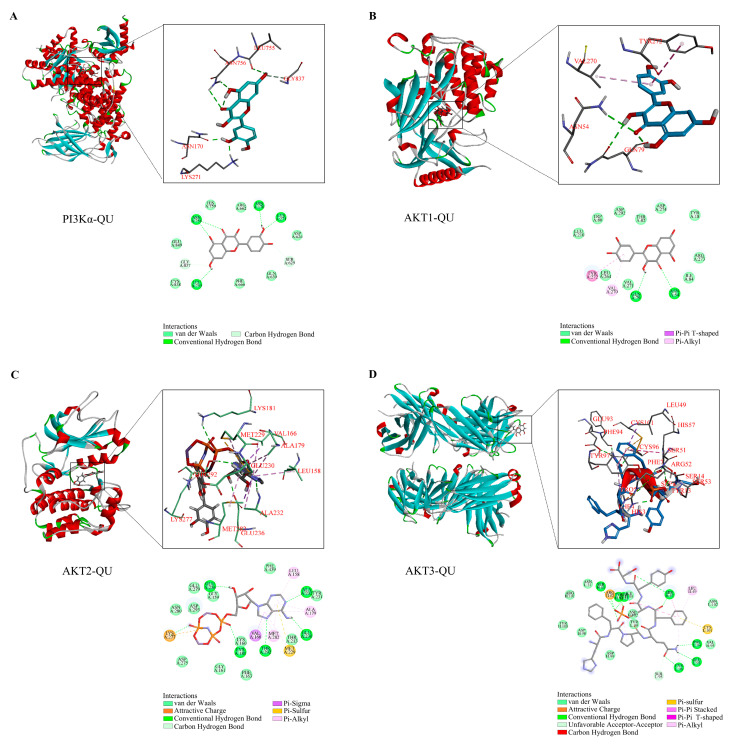
Molecular docking analysis of quercetin with PI3K and AKT family proteins. (**A**–**D**) Molecular docking models of quercetin with PI3K, AKT1, AKT2, and AKT3, respectively.

To further validate the interaction between QU and key signaling molecules, molecular docking analysis was performed using QU as the ligand. QU exhibited strong binding affinities with PI3K, AKT1, AKT2, and AKT3, with binding energies below −7 kcal/mol (Figure 5A–D) [27]. Among these interactions, QU showed the lowest binding energy with PI3K (−28.7689 kcal/mol) and a strong binding interaction with AKT1 (−35.16 kcal/mol) (Supplementary Table S7). Multiple hydrogen bonds and van der Waals interactions were observed between QU and the target proteins.

Collectively, these network pharmacology and molecular docking analyses suggest that QU may exert therapeutic effects in comorbid insomnia with IBS-D by modulating PI3K/AKT-related signaling pathways.

### 3.6. Quercetin Attenuates Intestinal and Neuroinflammation in Comorbid Insomnia via the PI3K/AKT/NF-κB Signaling Pathway

To investigate whether quercetin (QU) modulates inflammatory responses in comorbid insomnia through the PI3K/AKT signaling pathway, the expression of pathway-related proteins in the hypothalamus was assessed by Western blotting, and mRNA levels of inflammatory cytokines in both hypothalamic and colonic tissues were quantified by RT-qPCR.

**Figure 6:**
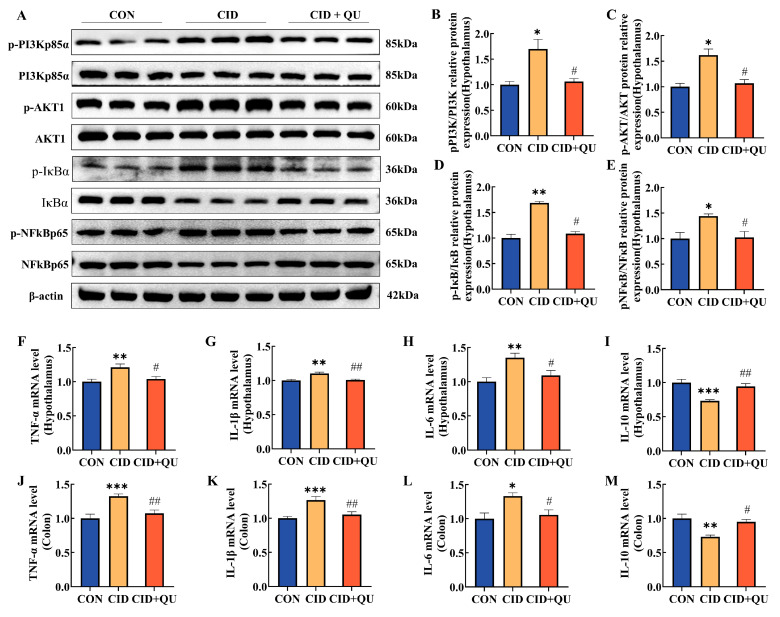
Effects of quercetin on the PI3K/AKT/NF-κB signaling pathway and inflammatory responses in CID mice. (**A**) Representative Western blot images showing the expression of PI3K/AKT pathway–related proteins in the hypothalamus, including p-PI3K, PI3K, p-AKT, and AKT. (**B**–**E**) Quantitative analysis of the relative protein expression levels of p-PI3K/PI3K, p-AKT/AKT, p-IκB/IκB, and p-NF-κB/NF-κB in the hypothalamus. (**F**–**I**) Relative mRNA expression levels of TNF-α, IL-1β, IL-6, and IL-10 in the hypothalamus. (**J**–**M**) Relative mRNA expression levels of TNF-α, IL-1β, IL-6, and IL-10 in colonic tissues. Data are presented as mean ± SEM (*n* = 6). * *p* < 0.05, ** *p* < 0.01, *** *p* < 0.001 vs. CON group; ^#^ *p* < 0.05, ^##^ *p* < 0.01.

In the hypothalamus, mice in the CID group exhibited significant activation of the PI3K/AKT pathway, as evidenced by markedly increased p-PI3K/PI3K and p-AKT/AKT ratios compared with the control (CON) group (both *p* < 0.05). QU treatment significantly reduced the phosphorylation levels of PI3K and AKT relative to the CID group, restoring them toward control levels (Figure 6A–C). Consistent with upstream pathway activation, downstream NF-κB signaling was also markedly enhanced in CID mice. The ratios of p-IκB/IκB and p-NF-κB/NF-κB were significantly elevated in the CID group compared with the CON group (*p* < 0.01 and *p* < 0.05, respectively). Administration of QU significantly suppressed the phosphorylation of both IκB and NF-κB, resulting in levels comparable to those observed in control mice (Figure 6D,E).

At the transcriptional level, CID mice exhibited pronounced neuroinflammatory responses in the hypothalamus. The mRNA expression levels of the proinflammatory cytokines TNF-α, IL-1β, and IL-6 were significantly increased, whereas the anti-inflammatory cytokine IL-10 was markedly decreased compared with the CON group (all *p* < 0.01). QU treatment significantly downregulated TNF-α, IL-1β, and IL-6 expression while upregulating IL-10 expression, yielding cytokine profiles similar to those of control mice (Figure 6F–I).

A parallel inflammatory pattern was observed in colonic tissues. Compared with the CON group, CID mice showed significantly increased mRNA levels of TNF-α, IL-1β, and IL-6, accompanied by a marked reduction in IL-10 expression (*p* < 0.01). QU administration significantly reversed these alterations, characterized by reduced proinflammatory cytokine expression and increased IL-10 levels, with no significant differences compared with the CON group (Figure 6J–M).

Collectively, these results indicate that quercetin treatment was accompanied by reduced activation of PI3K/AKT/NF-κB-related signaling and attenuation of both hypothalamic and colonic inflammatory responses in mice with comorbid insomnia-like and IBS-D-like phenotypes.

### 3.7. Recilisib Partially Reverses Quercetin-Associated Physiological, Sleep, and Molecular Improvements in CID Mice

To further interrogate whether PI3K/AKT-associated signaling participates in the protective effects of quercetin (QU) in this comorbid model, Recilisib, a pharmacological activator used to probe PI3K/AKT pathway involvement, was co-administered with QU in CID mice.

#### 3.7.1. Recilisib Reverses QU-Induced Improvements in Body Weight and Metabolic Intake

**Figure 7:**
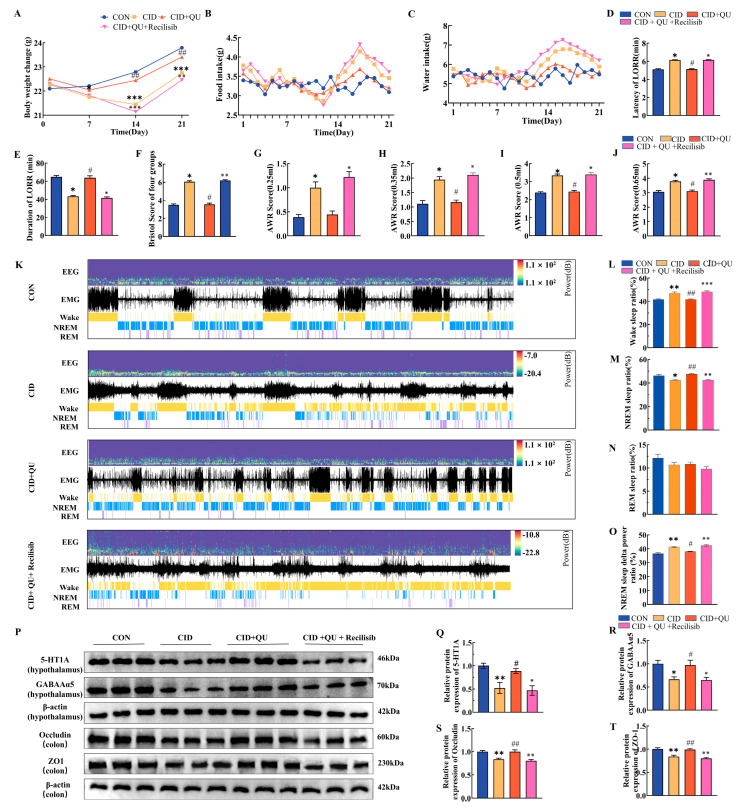
Recilisib abolishes quercetin-mediated improvements in physiological parameters, sleep architecture, visceral sensitivity, and intestinal–brain molecular markers in CID mice. (**A**) Body weight changes during the 21-day experimental period. (**B**,**C**) Daily food intake (**B**) and water intake (**C**) across treatment groups. (**D**,**E**) Sleep behavioral outcomes, including latency to loss of righting reflex (LORR) (**D**) and sleep duration (**E**). (**F**) Bristol Stool Scale scores assessing stool consistency. (**G**–**J**) Abdominal withdrawal reflex (AWR) scores under graded colorectal distension volumes (0.25, 0.35, 0.5, and 0.65 mL). (**K**) Representative EEG/EMG recordings illustrating wakefulness, NREM sleep, and REM sleep stages. (**L**–**N**) Quantification of wake ratio (**L**), NREM sleep ratio (**M**), and REM sleep ratio (**N**). (**O**) Relative delta power ratio during NREM sleep. (**P**) Representative Western blot images of hypothalamic 5-HT1A and GABA_ARα5, and colonic Occludin and ZO-1. (**Q**–**T**) Densitometric quantification of 5-HT1A (**Q**), GABA_ARα5 (**R**), Occludin (**S**), and ZO-1 (**T**), normalized to β-actin. Data are presented as mean ± SEM (*n* = 6). * *p* < 0.05, ** *p* < 0.01, *** *p* < 0.001 vs. CON group; ^#^ *p* < 0.05, ^##^ *p* < 0.01 vs. CID group; ^※^ *p* < 0.05, ^※※^ *p* < 0.01, ^※※※^ *p* < 0.001 vs. CID + QU group.

A significant interaction between treatment and time was observed for body weight changes across the experimental period. By day 21, QU-treated CID mice exhibited a significantly higher body weight compared with untreated CID mice (23.41 ± 0.29 g vs. 22.75 ± 0.29 g, *p* < 0.01). Notably, Recilisib co-treatment significantly reduced mean body weight relative to QU alone (22.46 ± 0.30 g vs. 23.41 ± 0.29 g, *p* < 0.01) (Figure 7A).

Consistent with these findings, CID mice displayed increased food and water intake compared with control (CON) mice, whereas QU administration reduced both parameters. Importantly, Recilisib co-administration restored food and water consumption compared with QU-treated mice (Figure 7B,C), indicating a reversal of QU-induced metabolic regulation.

#### 3.7.2. Effects of Recilisib on QU-Induced Improvements in Sleep Parameters

Sleep behavioral evaluation demonstrated that CID mice exhibited a significantly prolonged latency to loss of righting reflex (LORR) compared with controls (6.132 ± 0.027 min vs. 5.10 ± 0.07 min, *p* < 0.05). QU treatment markedly shortened LORR latency relative to CID mice (5.12 ± 0.06 min vs. 6.132 ± 0.027 min, *p* < 0.05).

In contrast, Recilisib co-treatment significantly prolonged induction latency compared with QU alone (6.14 ± 0.03 min vs. 5.12 ± 0.06 min, *p* < 0.05), suggesting that pharmacological activation of PI3K/AKT signaling abolishes QU-mediated sleep latency improvement (Figure 7D,E).

#### 3.7.3. Recilisib Worsens Gastrointestinal Function and Visceral Sensitivity in QU-Treated CID Mice

Gastrointestinal functional assessment using the Bristol Stool Scale revealed that CID mice produced significantly looser stools than CON mice (*p* < 0.05). QU treatment improved stool consistency, resulting in firmer stools compared with controls (*p* < 0.05). However, Recilisib administration significantly increased Bristol scores, reaching values comparable to the QU group (*p* < 0.05) (Figure 7F).

Visceral hypersensitivity analysis further showed significant AWR responses under graded colorectal distension in all groups. Although QU did not markedly reduce AWR scores, Recilisib co-treatment consistently increased AWR scores compared with both CON and QU groups at all stimulus intensities, whereas no significant differences were observed in CID mice (*p* > 0.05) (Figure 7G–J). These results indicate that PI3K/AKT activation aggravates visceral sensitivity despite QU treatment.

#### 3.7.4. Recilisib Disrupts QU-Induced Normalization of Sleep Architecture and Neuroinflammatory Signaling

EEG/EMG monitoring revealed distinct group-dependent alterations in arousal dynamics. CID mice displayed a significantly higher arousal rate than CON mice (0.47 ± 0.01 vs. 0.42 ± 0.00, *p* < 0.01), whereas QU treatment reduced arousal to control-like levels (0.42 ± 0.00 vs. 0.47 ± 0.01, *p* < 0.01).

Importantly, Recilisib markedly increased arousal relative to QU-treated mice (0.48 ± 0.01 vs. 0.42 ± 0.00, *p* < 0.001), with no significant difference compared with CID mice (Figure 7K,L).

Sleep stage distribution analysis demonstrated that CID mice exhibited a reduced proportion of NREM sleep compared with controls (0.42 ± 0.01 vs. 0.46 ± 0.01, *p* < 0.05). QU significantly increased NREM proportion (0.48 ± 0.01 vs. 0.42 ± 0.01, *p* < 0.01), whereas Recilisib co-treatment reduced NREM sleep compared with QU alone (0.42 ± 0.00 vs. 0.48 ± 0.01, *p* < 0.01) (Figure 7M). REM sleep proportions remained unchanged among groups (*p* > 0.05) (Figure 7N).

CID mice also exhibited elevated NREM delta power compared with CON mice (0.414 ± 0.01 vs. 0.37 ± 0.01, *p* < 0.01). QU reduced delta activity (0.38 ± 0.00 vs. 0.414 ± 0.01, *p* < 0.05), whereas Recilisib increased it again compared with QU-treated mice (0.42 ± 0.01 vs. 0.38 ± 0.00, *p* < 0.01) (Figure 7O).

At the molecular level, CID mice exhibited reduced hypothalamic expression of 5-HT1A (0.52 ± 0.12 vs. 1.00 ± 0.06; *p* < 0.01) and GABA_ARα5 (0.67 ± 0.05 vs. 1.00 ± 0.07; *p* < 0.05), together with decreased colonic Occludin and ZO-1 expression, indicating impaired neurotransmitter balance and intestinal barrier integrity. QU restored these proteins, whereas Recilisib reversed QU-mediated upregulation (Figure 7P–T).

**Figure 8:**
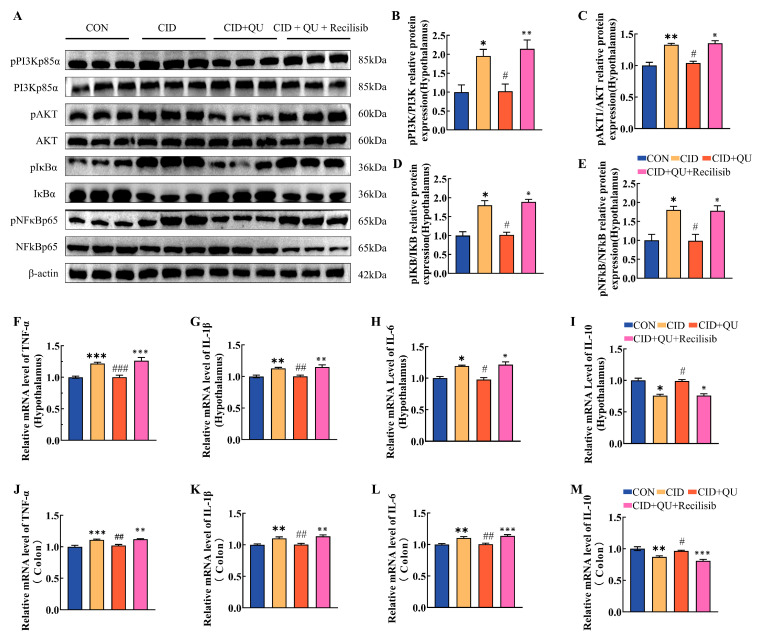
Recilisib restores PI3K/AKT/NF-κB activation and inflammatory cytokine expression suppressed by quercetin in CID mice. (**A**) Representative Western blot images showing phosphorylated and total PI3K, AKT, IκB, and NF-κB p65 in hypothalamic tissues. (**B**–**E**) Quantification of signaling activation ratios: p-PI3K/PI3K (**B**), p-AKT/AKT (**C**), p-IκB/IκB (**D**), and p-NF-κB/NF-κB (**E**). (**F**–**I**) Relative mRNA expression of TNF-α (**F**), IL-1β (**G**), IL-6 (**H**), and IL-10 (**I**) in hypothalamic tissues. (**J**–**M**) Relative mRNA expression of TNF-α (**J**), IL-1β (**K**), IL-6 (**L**), and IL-10 (**M**) in colonic tissues. Data are presented as mean ± SEM (*n* = 6). * *p* < 0.05, ** *p* < 0.01, *** *p* < 0.001 vs. CON group; ^#^ *p* < 0.05, ^##^ *p* < 0.01, ^###^ *p* < 0.01 vs. CID group; ^※^ *p* < 0.05; ^※※^ *p* < 0.01; ^※※※^ *p* < 0.001 vs. CID + QU group.

Mechanistically, CID mice exhibited enhanced activation of the PI3K/AKT signaling cascade, as reflected by elevated p-PI3K/PI3K (*p* < 0.05) and p-AKT/AKT ratios (*p* < 0.01). QU treatment significantly suppressed these phosphorylation levels (*p* < 0.05), whereas Recilisib co-administration reinstated PI3K/AKT activation (Figure 8A–C). Consistently, CID-associated NF-κB signaling and downstream proinflammatory cytokine expression were attenuated by QU but restored by Recilisib (Figure 8D–M). Importantly, this molecular reactivation was accompanied by the loss of QU-induced improvements in sleep architecture, visceral sensitivity, and intestinal–brain axis markers, including reduced hypothalamic 5-HT1A/GABA_ARα5 expression and impaired colonic tight junction integrity (Occludin and ZO-1) (Figure 7K–T).

At the molecular level, CID mice exhibited increased phosphorylation of PI3K, AKT, IκB, and NF-κB p65 in hypothalamic tissue, together with a pro-inflammatory cytokine profile in both the hypothalamus and colon. QU treatment attenuated these signaling and inflammatory changes, whereas Recilisib co-administration partially restored them. This molecular reactivation was accompanied by loss of several QU-associated improvements in sleep architecture, intestinal function, hypothalamic 5-HT1A/GABA_ARα5 expression, and colonic tight-junction protein expression. Taken together, these findings support the involvement of PI3K/AKT-associated inflammatory signaling in QU-mediated protection, although they do not by themselves establish pathway exclusivity.

## 4. Discussion

This study combined bioinformatics screening, network pharmacology, molecular docking, and in vivo validation to investigate the therapeutic potential of quercetin in a composite murine model exhibiting both insomnia-like and IBS-D-like phenotypes. The main findings were that quercetin improved sleep-associated behavioral and electrophysiological indices, ameliorated diarrhea-like stool changes and visceral hypersensitivity, restored colonic ZO-1 and Occludin expression, reduced inflammatory cytokine expression in the hypothalamus and colon, and was accompanied by reduced phosphorylation of PI3K, AKT, IκB, and NF-κB p65 in hypothalamic tissue. In addition, the partial reversal induced by Recilisib supports the involvement of PI3K/AKT-associated signaling in these protective effects. Accordingly, the present data support quercetin as a multitarget modulator of gut–brain dysfunction rather than as a single-pathway agent.

A key mechanistic observation of this work is that QU suppresses inflammatory activation through inhibition of PI3K/AKT-driven NF-κB signaling. This is consistent with previous evidence showing that polyphenolic compounds modulate PI3K/AKT pathways to exert barrier-protective and anti-inflammatory effects [37,38]. NF-κB is a central transcriptional regulator of proinflammatory mediators such as TNF-α and IL-6, which are elevated in IBS-D and have been implicated in visceral hypersensitivity and sleep disruption [39,40]. In our model, QU reduced diarrhea frequency and visceral pain responses while concurrently suppressing NF-κB activation, extending prior reports that flavonoids, including quercetin, mitigate inflammatory cytokine release across diverse pathological contexts [41,42]. An important strength of the present work is that the experimental model reproduced convergent phenotypes relevant to both sides of the gut–brain axis. Sleep disturbance was supported by prolonged sleep latency, altered EEG/EMG-derived sleep architecture, increased arousal, and abnormal NREM-related delta activity, whereas IBS-D-like manifestations were supported by loose stool output, increased AWR scores under graded colorectal distension, and reduced tight-junction protein expression. We agree, however, that the model should be interpreted cautiously. Because direct intestinal permeability assays were not performed, the present study does not claim exhaustive validation of every hallmark feature of IBS-D. Rather, our data indicate that the model recapitulates major sleep-related and gut-related phenotypic dimensions of CID + IBS-D comorbidity. This interpretation is consistent with prior preclinical work showing that stress-based paradigms combined with laxative or chemical stimulation can reproduce key IBS-D-related features, including altered bowel function, visceral hypersensitivity, and barrier-associated abnormalities [29].

Notably, we observed an elevation in δ-wave power during NREM sleep in CID mice, which differs from the reduced slow-wave activity typically reported in primary insomnia [43,44]. Under conditions of visceral hypersensitivity and chronic low-grade inflammation, comorbid insomnia may involve maladaptive remodeling of sleep homeostasis through immune–neural circuits, resulting in altered slow-wave dynamics [45,46,47]. Emerging evidence suggests that microglia-derived TNF-α can influence synaptic GABA_A receptor distribution and reshape NREM oscillatory activity, providing neurobiological support for an “inflammation–slow-wave remodeling” mechanism [48]. However, further studies are required to determine whether this electrophysiological phenotype represents a distinct signature of IBS-D-associated insomnia.

Mechanistically, our findings are most consistent with a model in which quercetin attenuates gut–brain inflammatory activation and improves both intestinal and sleep-related phenotypes in parallel. The observed reduction in hypothalamic p-PI3K/PI3K, p-AKT/AKT, p-IκB/IκB, and p-p65/p65 ratios, together with decreased TNF-α, IL-1β, and IL-6 expression and increased IL-10 expression, supports suppression of inflammatory signaling associated with the PI3K/AKT/NF-κB axis. However, the mechanistic scope of the present data should not be overstated. Although p65 phosphorylation was assessed, NF-κB nuclear translocation was not directly measured by subcellular fractionation or immunostaining. Likewise, the Recilisib experiment provides pharmacological support for pathway involvement, but not definitive proof of pathway exclusivity. This more conservative interpretation is appropriate given that pharmacological activators can have context-dependent off-target effects and that other signaling axes, such as MAPK or JAK/STAT, were not examined in this study [49].

Another important issue concerns the relative contribution of central versus peripheral mechanisms. In the present study, hypothalamic signaling and neurotransmission-related proteins were directly evaluated, and these central readouts changed in parallel with colonic inflammatory and barrier-related indices. Therefore, our findings support integrated modulation of a gut–brain inflammatory network. Nevertheless, the current data do not establish whether the sleep benefit of quercetin reflects direct central nervous system exposure or is secondary to reduced intestinal inflammation and improved barrier integrity. This distinction is particularly relevant because quercetin is known to have limited oral bioavailability, and brain exposure after oral dosing may be restricted. Since neither blood–brain barrier permeability nor plasma/brain concentrations were measured here, direct CNS pharmacokinetic action cannot be concluded from the current dataset [50].

Sleep-related outcomes in this study were consistent with barrier restoration. QU shortened sleep latency, increased total sleep time, reduced arousals, normalized NREM proportions, and lowered δ-wave power. These improvements coincided with the recovery of 5-HT1A/GABAARα5 signaling and increased ZO-1 and Occludin expression. Together, these data suggest a translatable pathway: epithelial repair reduces systemic inflammatory tension, which in turn stabilizes sleep architecture [36]. To maintain mechanistic interpretability, microbiota-related analyses (e.g., 16S rRNA sequencing or short-chain fatty acid profiling) were not included, as the present work focused on the directly targetable “barrier–inflammation–sleep” axis. Additionally, sedative controls were avoided to prevent confounding upstream immune and epithelial readouts. Recilisib, therefore, served as a pharmacological tool to interrogate pathway specificity. A single literature-supported QU dose was applied to establish efficacy [32,51,52,53], and future investigations may incorporate dose–response designs.

Several limitations should be acknowledged. First, although the model displayed robust IBS-D-like and insomnia-like phenotypes, direct intestinal permeability testing was not performed. Second, NF-κB nuclear translocation, brain bioavailability, and tissue pharmacokinetics of quercetin were not assessed. Third, only male mice were included in this exploratory mechanistic study. This design reduced biological variability during the initial pathway-focused investigation, but it also limits generalizability, particularly because both IBS and insomnia exhibit important sex-related differences in prevalence, symptom expression, and neuroimmune regulation. Fourth, Recilisib was used as a pharmacological tool to probe PI3K/AKT pathway involvement, but contributions from broader signaling crosstalk cannot be excluded. Finally, the mouse dose used in this study should be interpreted with caution in translational settings; based on standard body-surface-area conversion, 100 mg/kg/day in mice corresponds to an approximate human equivalent dose of 8.1 mg/kg/day, or about 486 mg/day for a 60 kg adult. Human studies in non-IBS conditions suggest that formulated oral quercetin at approximately 500 mg/day is tolerated and may show biological or clinical activity [54], but direct clinical evidence in CID + IBS-D remains lacking. but exposure-confirmation studies are still needed [50].

## 5. Conclusions

This study evaluated the effects of quercetin in a composite murine model displaying both insomnia-like and diarrhea-predominant irritable bowel syndrome (IBS-D)-like phenotypes. Quercetin improved sleep-related behavioral and electrophysiological parameters, alleviated diarrhea-like stool alterations and visceral hypersensitivity, restored colonic tight-junction protein expression, and attenuated inflammatory responses in both hypothalamic and colonic tissues.

These protective effects were accompanied by modulation of PI3K/AKT/NF-kB-related signaling, and were partially reversed by Recilisib, supporting the involvement of this pathway in quercetin-mediated gut–brain protection. However, the present findings do not establish pathway exclusivity or direct causality, and additional studies are needed to further define the relative contributions of central and peripheral mechanisms.

Overall, quercetin may represent a promising multitarget candidate for gut–brain axis-related comorbid conditions involving IBS-D-like and insomnia-like features. Nevertheless, further investigations are required to clarify sex-dependent effects, pharmacokinetic exposure, dose–response relationships, and clinical translational relevance before its therapeutic potential in human populations can be fully assessed.

## Data Availability

The datasets generated and analyzed during the current study are available from the corresponding author upon reasonable request.

## Supplementary Materials

The following supporting information can be downloaded at https://www.mdpi.com/article/10.3390/biomedicines14030692/s1: Figure S1: Schematic diagram of the experimental design and group allocation.; Table S1: The 344 overlapping differentially expressed genes between CID and IBS-D; Figure S2: Volcano plots of differentially expressed genes in the insomnia and IBS-D datasets; Table S2: Overlapping genes identified by WGCNA analysis between CID and IBS-D. Figure S3: Determination of the soft-thresholding power in weighted gene co-expression network analysis (WGCNA); Table S3: The 351 integrated overlapping genes after deduplication between CID and IBS-D. Table S4. List of 43 small-molecule compounds enriched based on the integrated overlapping genes. Table S5. Protein–protein interaction (PPI) network constructed from the 43 overlapping targets. Table S6. Gene Ontology (GO) enrichment analysis and KEGG pathway enrichment analysis of the 43 common driver genes. Table S7. Molecular docking binding energies of quercetin (QU) with PI3K, AKT1, AKT2, and AKT3.

## Abbreviations

The following abbreviations are used in this manuscript:

AKT	Protein kinase B
AWR	Abdominal withdrawal reflex
CDGs	Common driver genes
cDNA	Complementary DNA
CI	Chronic insomnia
CID	Chronic insomnia disorder
CON	Control group
DEGs	Differentially expressed genes
DSigDB	Drug Signature Database
EEG	Electroencephalography
EMG	Electromyography
GEO	Gene Expression Omnibus
GO	Gene Ontology
IBS	Irritable bowel syndrome
IBS-D	Diarrhea-predominant irritable bowel syndrome
IL-1β	Interleukin-1β
IL-6	Interleukin-6
IκB	Inhibitor of nuclear factor kappa B
KEGG	Kyoto Encyclopedia of Genes and Genomes
LORR	Loss of righting reflex
ME	Module eigengenes
mRNA	Messenger RNA
NCBI	National Center for Biotechnology Information
NF-κB	Nuclear factor-kappa B
p-AKT	Phosphorylated protein kinase B
PI3K	Phosphatidylinositol 3-kinase
p-IκB	Phosphorylated inhibitor of nuclear factor kappa B
p-NF-κB	Phosphorylated nuclear factor kappa B
PPI	Protein–protein interaction
p-PI3K	Phosphorylated phosphatidylinositol 3-kinase
QU	Quercetin
RT-qPCR	Reverse transcription-quantitative polymerase chain reaction.
TNF-α	Tumor necrosis factor-alpha
WB	Western blotting
WGCNA	Weighted gene co-expression network analysis.

## References

[1] Benjafield A.V., Sert Kuniyoshi F.H., Malhotra A., Martin J.L., Morin C.M., Maurer L.F., Cistulli P.A., Pepin J.L., Wickwire E.M. (2025). Estimation of the global prevalence and burden of insomnia: A systematic literature review-based analysis. Sleep Med. Rev..

[2] Roth T. (2009). Comorbid insomnia: Current directions and future challenges. Am. J. Manag. Care.

[3] Wang B., Duan R., Duan L. (2018). Prevalence of sleep disorder in irritable bowel syndrome: A systematic review with meta-analysis. Saudi J. Gastroenterol..

[4] Lovell R.M., Ford A.C. (2012). Global Prevalence of and Risk Factors for Irritable Bowel Syndrome: A Meta-analysis. Clin. Gastroenterol. Hepatol..

[5] Enck P., Aziz Q., Barbara G., Farmer A.D., Fukudo S., Mayer E.A., Niesler B., Quigley E.M.M., Rajilić-Stojanović M., Schemann M. (2016). Irritable bowel syndrome. Nat. Rev. Dis. Primers.

[6] Lembo A., Sultan S., Chang L., Heidelbaugh J.J., Smalley W., Verne G.N. (2022). AGA Clinical Practice Guideline on the Pharmacological Management of Irritable Bowel Syndrome with Diarrhea. Gastroenterology.

[7] Chey W.D., Keefer L., Whelan K., Gibson P.R. (2021). Behavioral and Diet Therapies in Integrated Care for Patients with Irritable Bowel Syndrome. Gastroenterology.

[8] Moloney R.D., O’Mahony S.M., Dinan T.G., Cryan J.F. (2015). Stress-induced visceral pain: Toward animal models of irritable-bowel syndrome and associated comorbidities. Front. Psychiatry.

[9] Bashashati M., Moossavi S., Cremon C., Barbaro M.R., Moraveji S., Talmon G., Rezaei N., Hughes P.A., Bian Z.X., Choi C.H. (2018). Colonic immune cells in irritable bowel syndrome: A systematic review and meta-analysis. Neurogastroenterol. Motil..

[10] Langfelder P., Horvath S. (2008). WGCNA: An R package for weighted correlation network analysis. BMC Bioinform..

[11] Boots A.W., Haenen G.R.M.M., Bast A. (2008). Health effects of quercetin: From antioxidant to nutraceutical. Eur. J. Pharmacol..

[12] Li Y., Yao J., Han C., Yang J., Chaudhry M.T., Wang S., Liu H., Yin Y. (2016). Quercetin, Inflammation and Immunity. Nutrients.

[13] Davis S., Meltzer P.S. (2007). GEOquery: A bridge between the Gene Expression Omnibus (GEO) and BioConductor. Bioinformatics.

[14] Ritchie M.E., Phipson B., Wu D., Hu Y., Law C.W., Shi W., Smyth G.K. (2015). limma powers differential expression analyses for RNA-sequencing and microarray studies. Nucleic Acids Res..

[15] Wickham H., François R., Henry L., Müller K., Vaughan D. (2023). dplyr: A Grammar of Data Manipulation. https://CRAN.R-project.org/package=dplyr.

[16] Hao D., Yang X., Li Z., Xie B., Feng Y., Liu G., Ren X. (2025). Screening core genes for minimal change disease based on bioinformatics and machine learning approaches. Int. Urol. Nephrol..

[17] Kuleshov M.V., Jones M.R., Rouillard A.D., Fernandez N.F., Duan Q., Wang Z., Koplev S., Jenkins S.L., Jagodnik K.M., Lachmann A. (2016). Enrichr: A comprehensive gene set enrichment analysis web server 2016 update. Nucleic Acids Res..

[18] Shannon P., Markiel A., Ozier O., Baliga N.S., Wang J.T., Ramage D., Amin N., Schwikowski B., Ideker T. (2003). Cytoscape: A software environment for integrated models of biomolecular interaction networks. Genome Res..

[19] Li X., Miao F., Xin R., Tai Z., Pan H., Huang H., Yu J., Chen Z., Zhu Q. (2023). Combining network pharmacology, molecular docking, molecular dynamics simulation, and experimental verification to examine the efficacy and immunoregulation mechanism of FHB granules on vitiligo. Front. Immunol..

[20] Xue Q.Q., Liu C.H., Li Y. (2024). Decoding the anti-hypertensive mechanism of alpha-mangostin based on network pharmacology, molecular docking and experimental validation. Mol. Med..

[21] Szklarczyk D., Gable A.L., Lyon D., Junge A., Wyder S., Huerta-Cepas J., Simonovic M., Doncheva N.T., Morris J.H., Bork P. (2019). STRING v11: Protein-protein association networks with increased coverage, supporting functional discovery in genome-wide experimental datasets. Nucleic Acids Res..

[22] Liu X., Yu Y., Wu Y., Luo A., Yang M., Li T., Li T., Mao B., Chen X., Fu J. (2023). A systematic pharmacology-based in vivo study to reveal the effective mechanism of Yupingfeng in asthma treatment. Phytomedicine.

[23] Shi S., Zhang M., Xie W., Ju P., Chen N., Wang F., Lyu D., Wang M., Hong W. (2023). Sleep deprivation alleviates depression-like behaviors in mice via inhibiting immune and inflammatory pathways and improving neuroplasticity. J. Affect. Disord..

[24] Li N., Tan S., Wang Y., Deng J., Wang N., Zhu S., Tian W., Xu J., Wang Q. (2023). Akkermansia muciniphila supplementation prevents cognitive impairment in sleep-deprived mice by modulating microglial engulfment of synapses. Gut Microbes.

[25] Dong Y.J., Jiang N.H., Zhan L.H., Teng X., Fang X., Lin M.Q., Xie Z.Y., Luo R., Li L.Z., Li B. (2021). Soporific effect of modified Suanzaoren Decoction on mice models of insomnia by regulating Orexin-A and HPA axis homeostasis. Biomed. Pharmacother..

[26] Yasugaki S., Liu C.-Y., Kashiwagi M., Kanuka M., Honda T., Miyata S., Yanagisawa M., Hayashi Y. (2019). Effects of 3 Weeks of Water Immersion and Restraint Stress on Sleep in Mice. Front. Neurosci..

[27] Yuan Z., Zhang C., Peng X., Shu L., Long C., Tan Z. (2020). Intestinal microbiota characteristics of mice treated with *Folium senna* decoction gavage combined with restraint and tail pinch stress. 3 Biotech.

[28] Zhao Y., Luo D.-N., Chen Y., Huang C., Zhou S.-Y. (2017). Dose-effect and time-effect relationship of chronic restraint stress c ombined with senna extract gavage in inducing diarrhea-predominant irr itable bowel syndrome in rats. WCJD.

[29] Chen Q., Zhang H., Sun C.Y., He Q.Y., Zhang R.R., Luo B.F., Zhou Z.H., Chen X.F. (2023). Evaluation of two laboratory model methods for diarrheal irritable bowel syndrome. Mol. Med..

[30] Pei W., Xie C., Li J., Jiang H., Shi L., Yun Y., Xue X., Zhao X. (2023). Effect of Tongxie Anchang Decoction on visceral hypersensitivity and gut microbiota in diarrhea-predominant irritable bowel syndrome mice. Chin. J. Integr. Tradit. West. Med. Dig..

[31] Paula P.-C., Angelica Maria S.-G., Luis C.-H., Gloria Patricia C.-G. (2019). Preventive Effect of Quercetin in a Triple Transgenic Alzheimer’s Disease Mice Model. Molecules.

[32] Lv C., Hong T., Yang Z., Zhang Y., Wang L., Dong M., Zhao J., Mu J., Meng Y. (2012). Effect of Quercetin in the 1-Methyl-4-phenyl-1, 2, 3, 6-tetrahydropyridine-Induced Mouse Model of Parkinson’s Disease. Evid. Based Complement. Altern. Med..

[33] Cao J.X., Zhang Q.Y., Cui S.Y., Cui X.Y., Zhang J., Zhang Y.H., Bai Y.J., Zhao Y.Y. (2010). Hypnotic effect of jujubosides from *Semen Ziziphi Spinosae*. J. Ethnopharmacol..

[34] Lewis S.J., Heaton K.W. (1997). Stool form scale as a useful guide to intestinal transit time. Scand. J. Gastroenterol..

[35] Zhao Q., Yang W.R., Wang X.H., Li G.Q., Xu L.Q., Cui X., Liu Y., Zuo X.L. (2019). Clostridium butyricum alleviates intestinal low-grade inflammation in TNBS-induced irritable bowel syndrome in mice by regulating functional status of lamina propria dendritic cells. World J. Gastroenterol..

[36] Wu Z., Liu L., Li L., Cao X., Jia W., Liao X., Zhao Z., Qi H., Fan G., Lu H. (2023). Oral nano-antioxidants improve sleep by restoring intestinal barrier integrity and preventing systemic inflammation. Natl. Sci. Rev..

[37] Jamieson P.E., Carbonero F., Stevens J.F. (2023). Dietary (poly)phenols mitigate inflammatory bowel disease: Therapeutic targets, mechanisms of action, and clinical observations. Curr. Res. Food Sci..

[38] Zhu W., Gong A., Zhang B., Cheng H., Huang L., Wu X., Zhang D., Dai W., Li S., Xu H. (2025). The Chronobiological and Neuroprotective Mechanisms of Resveratrol in Improving Sleep. Mediat. Inflamm..

[39] Liu X., Huang Y., Wang Y., Lin C., Xu B., Zeng Y., Chen P., Huang Y., Liu X. (2025). Association between gastrointestinal symptoms and insomnia among healthcare workers: A cross-sectional study. Sci. Rep..

[40] Zhou Q., Yang L., Verne M.L., Zhang B.B., Fields J., Verne G.N. (2023). Catechol-O-Methyltransferase Loss Drives Cell-Specific Nociceptive Signaling via the Enteric Catechol-O-Methyltransferase/microRNA-155/Tumor Necrosis Factor α Axis. Gastroenterology.

[41] Das D., Banerjee A., Mukherjee S., Maji B.K. (2024). Quercetin inhibits NF-kB and JAK/STAT signaling via modulating TLR in thymocytes and splenocytes during MSG-induced immunotoxicity: An in vitro approach. Mol. Biol. Rep..

[42] Chaudhary S., Sharma S., Fuloria S., Sharma P.K. (2025). Anti-Inflammatory and Anti-Arthritis Activity of Quercetin: A Comprehensive Review. Curr. Rheumatol. Rev..

[43] Riemann D., Spiegelhalder K., Feige B., Voderholzer U., Berger M., Perlis M., Nissen C. (2010). The hyperarousal model of insomnia: A review of the concept and its evidence. Sleep Med. Rev..

[44] Hogan S.E., Delgado G.M., Hall M.H., Nimgaonkar V.L., Germain A., Buysse D.J., Wilckens K.A. (2020). Slow-oscillation activity is reduced and high frequency activity is elevated in older adults with insomnia. J. Clin. Sleep. Med..

[45] Krueger J.M. (2008). The role of cytokines in sleep regulation. Curr. Pharm. Des..

[46] Imeri L., Opp M.R. (2009). How (and why) the immune system makes us sleep. Nat. Rev. Neurosci..

[47] Besedovsky L., Lange T., Born J. (2012). Sleep and immune function. Pflügers Arch..

[48] Pinto M.J., Bizien L., Fabre J.M.J., Ðukanović N., Lepetz V., Henderson F., Pujol M., Sala R.W., Tarpin T., Popa D. (2024). Microglial TNFα controls daily changes in synaptic GABAARs and sleep slow waves. J. Cell Biol..

[49] Kang A.D., Cosenza S.C., Bonagura M., Manair M., Reddy M.V., Reddy E.P. (2013). ON01210.Na (Ex-RAD®) mitigates radiation damage through activation of the AKT pathway. PLoS ONE.

[50] Manta K., Papakyriakopoulou P., Nikolidaki A., Balafas E., Kostomitsopoulos N., Banella S., Colombo G., Valsami G. (2023). Comparative Serum and Brain Pharmacokinetics of Quercetin after Oral and Nasal Administration to Rats as Lyophilized Complexes with β-Cyclodextrin Derivatives and Their Blends with Mannitol/Lecithin Microparticles. Pharmaceutics.

[51] Yu X., Wang X., Liu X., Li F., Bao Y., Chai Y. (2024). The Mechanism of Relieving Diarrheal Irritable Bowel Syndrome Using Polyphenols from *Ribes nigrum* L. Based on a Network Pharmacology Analysis and 16S rRNA Sequencing. Foods.

[52] Niu Y.B., Yang Y.Y., Xiao X., Sun Y., Zhou Y.M., Zhang Y.H., Dong D., Li C.R., Wu X.L., Li Y.H. (2020). Quercetin prevents bone loss in hindlimb suspension mice via stanniocalcin 1-mediated inhibition of osteoclastogenesis. Acta Pharmacol. Sin..

[53] Long D., Zhao H., Wu X., Jin L., Ran Y., Hu Q., Zhao X., Zhang T., Tian M. (2026). Chinese bayberry (*Morella rubra* Lour.) seed improves DSS-induced inflammatory bowel disease by inhibiting inflammation and oxidative stress. J. Funct. Foods.

[54] Rondanelli M., Riva A., Petrangolini G., Gasparri C., Perna S. (2023). Two-month period of 500 mg lecithin-based delivery form of quercetin daily dietary supplementation counterbalances chronic fatigue symptoms: A double-blind placebo controlled clinical trial. Biomed. Pharmacother..

