# Supplementary figures and images for: Quercetin Ameliorates Comorbid Insomnia in Diarrhea-Predominant Irritable Bowel Syndrome via the PI3K/AKT/NF-κB Signaling Pathway
Source: Biomedicines. 2026 Mar 17;14(3):692. doi: 10.3390/biomedicines14030692 (PMC13023494; doi:10.3390/biomedicines14030692)

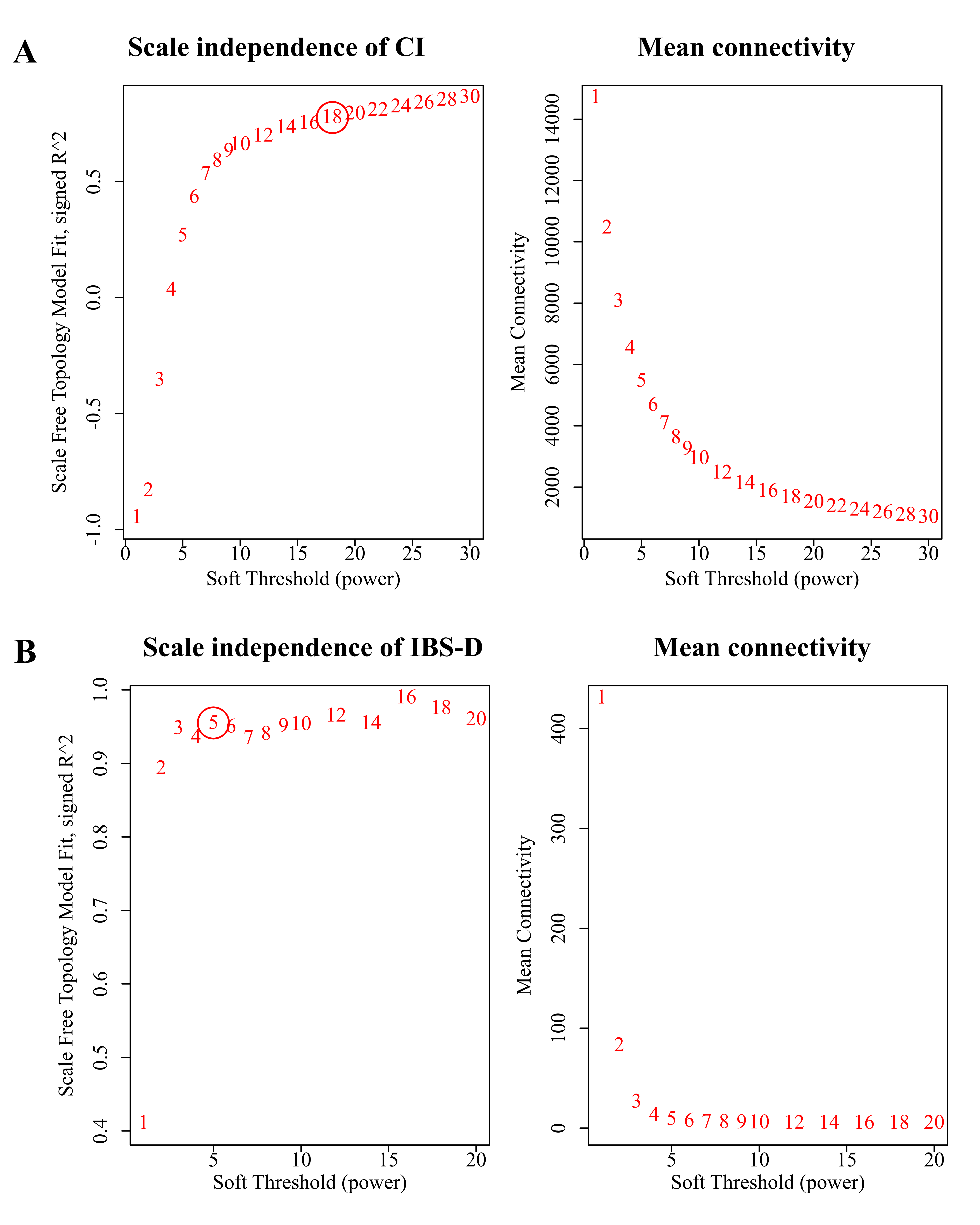

Supplement: Supplementary file 1 [file biomedicines-14-00692-s001.zip › Supplementary Figure S3.tif]
